# Elevational distribution patterns and drivers factors of fungal community diversity at different soil depths in the *Abies georgei* var. smithii forests on Sygera Mountains, southeastern Tibet, China

**DOI:** 10.3389/fmicb.2024.1444260

**Published:** 2024-08-09

**Authors:** Bo Zhang, Sijie Zhu, Jiangrong Li, Fangwei Fu, Liangna Guo, Jieting Li, Yibo Zhang, Yuzhuo Liu, Ganggang Chen, Gengxin Zhang

**Affiliations:** ^1^Research Institute of Tibet Plateau Ecology, Tibet Agricultural and Animal Husbandry University, Nyingchi, China; ^2^Key Laboratory of Forest Ecology in Tibet Plateau, Ministry of Education, Nyingchi, China; ^3^National Key Station of Field Scientific Observation and Experiment, Nyingchi, China; ^4^Key Laboratory of Alpine Vegetation Ecological Security in Tibet, Nyingchi, China; ^5^State Key Laboratory of Tibetan Plateau Earth System, Resources and Environment (TPESRE), Institute of Tibetan Plateau Research, Chinese Academy of Sciences, Beijing, China

**Keywords:** elevation, fungi, alpine forest, fungal diversity, co-occurrence network

## Abstract

**Introduction:**

Soil fungal communities play a crucial role in maintaining the ecological functions of alpine forest soil ecosystems. However, it is currently unclear how the distribution patterns of fungal communities in different soil layers of alpine forests will change along the elevational gradients.

**Material and methods:**

Therefore, Illumina MiSeq sequencing technology was employed to investigate fungal communities in three soil layers (0–10, 10–20, and 20–30 cm) along an elevational gradient (3500 m to 4300 m) at Sygera Mountains, located in Bayi District, Nyingchi City, Tibet.

**Results and discussion:**

The results indicated that: 1) Soil depth had a greater impact on fungal diversity than elevation, demonstrating a significant reduction in fungal diversity with increased soil depth but showing no significant difference with elevation changes in all soil layers. Within the 0–10 cm soil layer, both *Basidiomycota* and *Ascomycota* co-dominate the microbial community. However, as the soil depth increases to 10–20 and 20–30 cm soil layers, the *Basidiomycota* predominantly dominates. 2) Deterministic processes were dominant in the assembly mechanism of the 0–10 cm fungal community and remained unchanged with increasing elevation. By contrast, the assembly mechanisms of the 10–20 and 20–30 cm fungal communities shifted from deterministic to stochastic processes as elevation increased. 3) The network complexity of the 0–10 cm fungal community gradually increased with elevation, while that of the 10–20 and 20–30 cm fungal communities exhibited a decreasing trend. Compared to the 0–10 cm soil layer, more changes in the relative abundance of fungal biomarkers occurred in the 10–20 and 20–30 cm soil layers, indicating that the fungal communities at these depths are more sensitive to climate changes. Among the key factors driving these alterations, soil temperature and moisture soil water content stood out as pivotal in shaping the assembly mechanisms and network complexity of fungal communities. This study contributes to the understanding of soil fungal community patterns and drivers along elevational gradients in alpine ecosystems and provides important scientific evidence for predicting the functional responses of soil microbial ecosystems in alpine forests.

## 1 Introduction

With the intensification of global climate change, ecosystems are currently facing unprecedented challenges. Climate change not only affects species distribution but also profoundly impacts the structure and function of soil ecosystems. Soil, as a complex ecosystem, its health directly relates to the maintenance of global carbon cycling and biodiversity (Street et al., 2020; Suleiman et al., 2022). Alpine forest ecosystems, which are known for their distinctive ecological conditions and delicate balance, are regarded as among the most responsive to climatic variations (Donhauser and Frey, 2018; Verrall and Pickering, 2020). The escalating climate change is expected to have profound impacts on the vegetation composition and soil microbial communities of alpine forests, which has gained considerable attention from the scientific community (Hagedorn et al., 2019). Among the soil ecological functions, soil fungi as important decomposers play an indispensable role in plant growth, organic matter decomposition, and nutrient cycling (Basu et al., 2020; Li et al., 2022). In addition, soil fungi serve as a crucial driver of soil development and evolution in high-altitude ecosystems. Their traits not only facilitate carbon sequestration and accumulation in soils, enhancing the stability of soil carbon, but also effectively stabilize the soil organic carbon pool by improving carbon use efficiency, thereby strengthening the function of soil as a long-term carbon storage reservoir (Lin et al., 2023; Zhao et al., 2023). Changes in elevation often lead to alterations in various environmental factors, including temperature, humidity, and light exposure (Pan et al., 2021). Therefore, the potential connections between climate change and the ecological characteristics of soil fungi can be identified by conducting extensive research on the variational characteristics of soil fungal communities along elevational gradients (Lladó et al., 2017; Peay et al., 2017; Ni et al., 2018). However, current studies on soil fungal communities in alpine forests on the Qinghai–Tibet Plateau have primarily focused on surface soils, with a relatively limited understanding of the characteristics and response mechanisms of deep soil fungal communities (Chen J. et al., 2022; Fu et al., 2023a). Nonetheless, fungal communities in deep soil may possess even more unique ecological strategies and mechanisms to adapt to the extreme conditions in alpine forests, such as low temperatures, hypoxia, and nutrient limitations. Therefore, further exploration into the responses of fungal community interactions and assembly mechanisms in different soil layers of alpine forests to elevation changes can aid in the prediction of climate change impacts on soil microbial ecological functions.

In macroecology, co-occurrence networks and modular pattern analysis play a key role as powerful tools for examining the interactions among organisms and the influence of the environment on the coexistence of biological communities (Cornell et al., 2023). Fungi do not exist as solitary entities; rather, they establish intricate symbiotic networks with bacteria and plants through indirect and direct interactions, playing a crucial role in the regulation of soil microbial community structures and ecosystem functions (Hallam and Mccutcheon, 2015). Key metrics in network analysis, such as the number of edges, nodes, and average degree, are widely used in the assessment of interactions and network complexity among microbial communities (de Vries et al., 2018; Fu et al., 2023a). Studies have shown that various environmental factors, including temperature, precipitation, and soil nutrients, influence interactions within microbial networks (Li L. et al., 2023; Wu et al., 2024). For instance, climate warming may enhance the complexity of microbial networks, thereby influencing the overall function of ecosystems (Wagg et al., 2019; Yuan et al., 2021). Conversely, the complexity of soil microbial networks may be reduced through precipitation, adversely impacting soil multifunctionality (Wang et al., 2023). Research has recently indicated a downward trend in the complexity of soil fungal networks with increasing elevation in alpine forest ecosystems on the Qinghai–Tibet Plateau (Chen W. et al., 2022). However, the current understanding of how soil microbial network complexity in different soil layers responds to environmental changes in the alpine forest ecosystems of the Qinghai-Tibet Plateau remains unclear.

The assembly of microbial communities is a complex process influenced by deterministic and stochastic ecological processes (Xun et al., 2019; Wu L. et al., 2022). Deterministic processes are primarily based on niche mechanisms (Chen et al., 2019), whereas stochastic processes mainly reflect random changes in the relative abundances of species (Dini-Andreote et al., 2015; Zhou and Ning, 2017). Community assembly is highly sensitive to environmental changes, and the relative contribution of deterministic and stochastic processes can uncover the response mechanisms of microbial communities to such changes (Jiao et al., 2021). Studies have shown significant variations in the assembly patterns of microbial communities across different environmental conditions. For example, in subtropical paddy soils, soil fungal community assembly is predominantly stochastic in surface soil, while deterministic processes such as dispersal become more dominant in deeper soil layers (Li et al., 2020). This finding indicates the existence of differences and associations in microbial characteristics across spatial distributions, which can influence ecosystem functions. Fungal community assembly shifts from deterministic to stochastic processes during the secondary succession of subtropical forests (Liu et al., 2021). In alpine forests of the Qinghai–Tibet Plateau, fungal community assembly is mainly deterministic (Fu et al., 2024) but primarily stochastic in alpine meadows (Wu M. H. et al., 2022; Li J. et al., 2023). Differences in the assembly patterns of soil fungal communities are observed across environments and ecosystems due to environmental factors and spatial distributions. However, understanding of how elevation drives these assembly patterns, particularly in alpine forest ecosystems that are highly sensitive to climate change, remains limited. Therefore, explaining the effects of elevation on fungal community assembly patterns in different soil layers of alpine forests is crucial for understanding biodiversity and ecosystem functions (Anthony et al., 2020; Jiao et al., 2022).

Sygera Mountain, which is located in the southeast of the Qinghai–Tibet Plateau, is a typical representative of alpine forest zones, displaying diverse forest resources and distinct vertical climatic characteristics. This mountain serves as a crucial window for studying alpine forest ecosystems. The dark coniferous cloud fir is the dominant tree species in this mountain, thereby serving a pivotal role in maintaining soil and water conservation, stabilizing alpine treelines, and facilitating forest carbon cycles (Castro et al., 2010; Liang et al., 2011). This study employs the elevational gradient of Sygera Mountain as a natural model to simulate the effects of climate change on fungal communities and comprehensively examine the impacts of climate change on soil fungi in alpine forests. Environmental factors undergo comprehensive changes as elevation increases, exerting high pressure on the survival of microorganisms. Simultaneously, a decrease in soil nutrients can be attributed to the increasing soil depth, and the supply of nutrients and oxygen in deep soil is already quite limited. As the elevation continues to rise, these limiting factors may further intensify, leading to a sharp increase in the survival pressure of fungal communities in deep soil and making their survival conditions increasingly severe. Based on these considerations, the following hypotheses are proposed: (1) soil fungal community diversity and network complexity will decrease significantly in all soil layers; (2) the stochastic assembly process of soil fungal communities will diminish with increasing environmental pressure elevation, and deterministic processes will impact fungal community structures at high elevations; (3) 10–20 and 20–30 cm soil layers fungal communities will be highly sensitive to elevational changes.

## 2 Materials and methods

### 2.1 Study area

**Figure 1:**
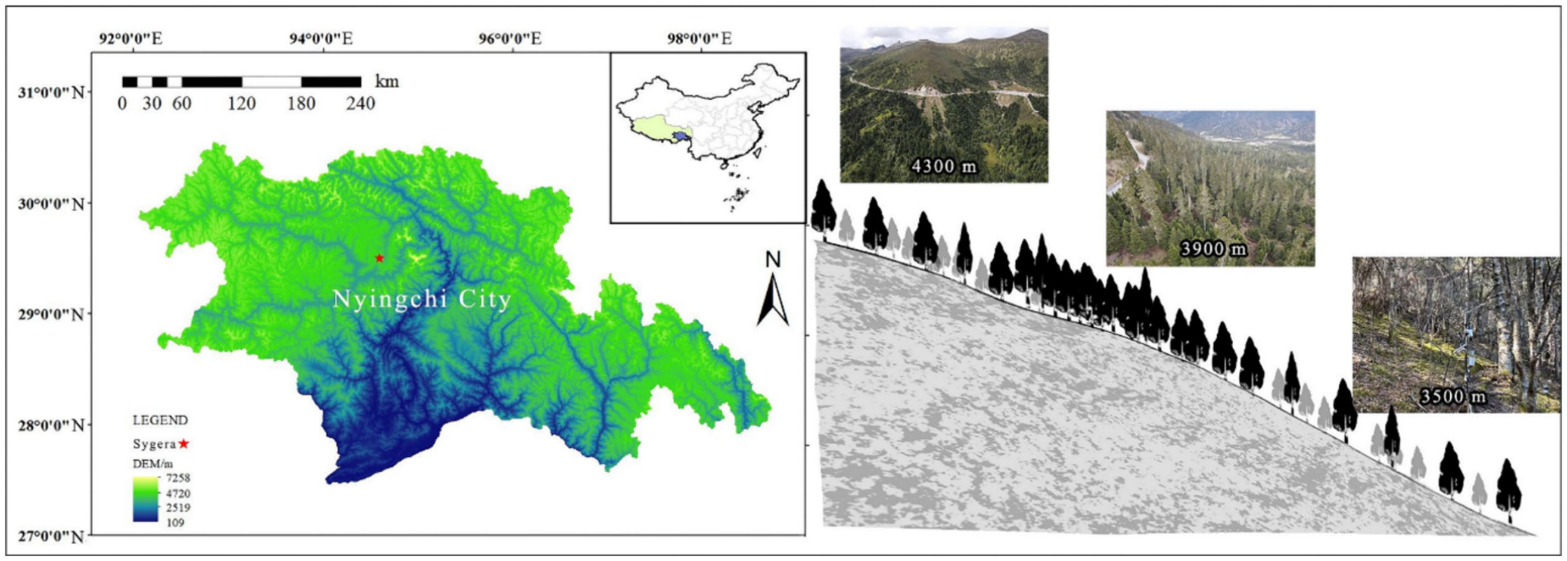
Distribution of sampling sites on Sygera Mountain.

Sygera Mountain is located southeast of Bayi District, Nyingchi City, in the southeast of the Tibet Autonomous Region. This mountain is the intersection of the southern extension of the Nyanqentanglha Mountains and the northern expansion of the Eastern Himalayas, with a geographic location of 29°10′–30°15′ N, 93°12′–95°35′ E (Figure 1). The elevation of the main peak is approximately 5,300 m. This region belongs to a humid mountainous warm temperate and semi-humid mountainous temperate climate, with distinct wet and dry seasons (Chen et al., 2023). The annual average temperature in this region is −0.73°C, the highest monthly (July) average temperature is 9.23°C, and the lowest monthly (January) average temperature is −13.98°C. The average annual precipitation is 1,134 mm, with the rainy season from June to September accounting for approximately 80% of the annual precipitation. The annual average relative humidity is 78.8%, and the annual sunshine duration is 1,151 h. The soil type is mountainous brown soil, and the research site is a mature primary forest of fir trees, distributed in the elevation range of 3,500–4,300 m. Table 1 shows the plot information.

**Table 1:** Basic information of *Abies georgei* var. smithii forest sample points.

Elevation (m)	Longitude and latitude	Soil type	Slope (°)	Aspect (°)	Tree height (m)	Tree diameter at breast height (DBH) (cm)	Crown density (%)	Annual temperature (°C)	Annual precipitation (mm)
3,500	94°43′31″E 29°41′54″N	Dark acidic Brownish soil	22	25	21.3 ± 1.6	37.4 ± 17.3	80	11.33	826.6
3,900	94°42′52″E 29°39′3″N	Dark acidic Brownish soil	27	155	15.4 ± 1.5	36.1 ± 14.5	85	4.40	867.1
4,300	94°41′42″E 29°36′52″N	Dark acidic Brownish soil	25	55	10.7 ± 1.2	34.6 ± 13.3	70	1.23	966.3

Annual average temperature and annual precipitation are the data of automatic weather stations in the sample area.

### 2.2 Sample site setup and soil sample collection

On June 22, 2021, soil sampling was conducted at three elevation gradients ranging from 3,500 m to 4,300 m in the Sygera Mountains in southeastern Tibet. Three 30 m × 30 m standard plots were established at each sampling elevation. Each sampling location was spaced 400 m apart. The five-point method (four corners and the center of the sample square) was used in the collection of soil samples from the following three depth layers: 0–10, 10–20, and 20–30 cm. The soil samples collected from each plot were mixed into one composite sample, and nine replicate samples were selected from each composite sample, yielding a total of 81 soil samples (three elevations, three sample plots set at each elevation, three samples per sample plot, and three samples of each soil layer). The collected soil samples were then placed in sealed bags and stored in ice boxes for transportation to the laboratory for subsequent analysis. After soil collection, the samples were sieved through a 1 mm soil sieve to remove roots, soil animals, and stones. Each sample was divided into two parts: one part was placed in a sterile bag and immediately cooled for DNA extraction (stored at −80°C). The remaining portion of the sample was air-dried and ground to determine its physicochemical properties.

### 2.3 Determination of soil physical and chemical properties

A soil carbon and nitrogen analyzer (Elementar Vario EL III, Germany) was used to measure soil total organic carbon (TOC) and soil total nitrogen (TN). Soil total phosphorus (TP) and soil available phosphorus (AP) were analyzed using the molybdenum–antimony spectrophotometric method (Song et al., 2022). Soil ammonium nitrogen (NH_4_^+^-N) and nitrate nitrogen (NO_3_^−^-N) were determined using a continuous flow analyzer with a 1 mol/L potassium chloride solution at a soil-to-solution ratio of 1:5 for extraction (AA3, SEAL Analytical, Germany). Soil available nitrogen (AN) was measured using the alkaline hydrolysis method. The soil pH was determined using a pH meter with a soil-to-water ratio of 1:2.5. Soil temperature sensors (S-TMB-MOO6) and soil moisture sensors (S-SMD-MOO5) were utilized to measure soil water content (SWC) and soil temperature (ST), respectively. In this study, meteorological data from January to December 2021 were collected, including soil temperature and moisture content at a depth of 0–30 cm, recorded every 10 min. The data logger used was the HOBO H21-USB, produced by Onset Computer Corporation in the United States. Meteorological data were downloaded once every month.

### 2.4 DNA extraction, amplicon sequencing, and bioinformatic analyses

Soil DNA extraction, PCR amplification, and Illumina sequencing were completed by Shanghai Majorbio Bio-Pharm Technology Co., Ltd. (Shanghai, China). Total DNA from soil samples was extracted using the QJ-Soil kit and all the extraction details are listed in the manufacturer’s instructions. The concentration and purity of DNA were measured using NanoDrop 2000 and 1% agarose gel electrophoresis, respectively. The internal transcribed spacer (ITS) regions of fungi were amplified using primers ITS1F (5′-CTTGGTCATTTAGAGTAA-3′) and ITS2R (5′-GCTGCGTTTCTTCATCGATGC-3′). The PCR mixture was prepared in a final volume of 20 μL, including 4 μL of 5 × FastPfu buffer, 2 μL of 2.5 mM of each dNTP, 0.4 μL of each primer (5 μM), 0.4 μL of FastPfu polymerase (Majorbio Bio-Pharm Technology Co., Ltd., Shanghai, China), 10 ng of template DNA, and 2.8 μL of ddH_2_O. The PCR reaction cycling involved an initial denaturation step at 94°C for 85 s, followed by 35 cycles of denaturation at 94°C for 35 s, annealing at 55°C for 55 s, and elongation at 72°C for 45 s, with a final elongation step at 72°C for 10 min. PCR products were extracted from a 2% agarose gel, purified using an AxyPrep DNA Gel Extraction Kit (Axygen Biosciences, Union City, CA, United States) according to the instructions of the manufacturer, and quantified using a Quantus^™^ Fluorometer (Promega, United States). Purified amplicons were mixed in equal molar proportions and subjected to paired-end sequencing on an Illumina MiSeq PE300 platform following the standard protocol of Majorbio Bio-Pharm Technology Co. Ltd. (Shanghai, China).

Raw reads without adapters from the MiSeq sequencer were assigned to different samples based on barcodes. Paired-end reads with at least a 50-bp overlap, and <5% mismatches were combined using flash (version 1.0.0). A threshold of average quality scores >30 over a 5-bp window size was used to trim the unqualified sequences using btrim (version 1.0.0). Any joined sequences with ambiguous bases and lengths <200 bp were discarded. Highquality sequences were clustered into operational taxonomic units (OTUs) with 97% similarity using uparse (version usearch v7.0.1001_i86), while the chimeras and singletons were discarded. Quantitative Insights Into Microbial Ecology (QIIME) version 1.9.1 (Caporaso et al., 2010) was used for sequence analysis, including quality control, taxonomic annotation, and generation of fungal operational taxonomic units (OTUs). Chimeras, single OTUs, and low-quality sequences were manually removed. USEARCH version 10.0 was used to identify OTUs at 97% similarity and cluster them using UPARSE version 7.0.1 (Edgar, 2013). Fungal ITS sequences were compared against the Silva version 132 (Quast et al., 2013) and Unite version 7.2 (Nilsson et al., 2019) databases, respectively.

### 2.5 Data statistics

The differences in fungal species composition in soils at different elevations were tested using a one-way ANOVA. The significance of these differences was determined using the least significant difference test at the 0.05 level. The Shapiro–Wilk and Levene’s tests were used to assess the normality of the data and homogeneity of variance, respectively. The α-diversity of the fungal community was characterized using the selected Shannon index. Nonmetric multidimensional scaling (NMDS) based on the Bray–Curtis distance algorithm was applied to analyze fungal diversity in the soil. Analysis of similarity (ANOSIM) was used to determine differences in fungal community composition in the soil, thereby judging the differences in soil fungal communities with elevation changes. RDA was used to detect the relationship between environmental variables and soil fungal communities. The linear discriminant analysis effect size (LEfSe) method^1^ was employed to detect potential biomarkers at multiple taxonomic levels using a linear discriminant analysis score threshold of >3.0 and an α-value of 0.05 for the factorial Kruskal–Wallis test. The neutral community model and normalized stochasticity ratio (NST) were set to 50% in R (Ning et al., 2019) to test the predominance of deterministic or stochastic processes in shaping the microbial community. The “NST” R package was used to calculate NST levels with 1,000 randomizations. An NST value <50% indicates the dominance of deterministic processes in the microbial community; otherwise, stochasticity is the dominant process. Network analysis was employed to investigate biological interactions between microbial populations and explore the mechanisms of OTU interactions in soil fungal communities following elevation changes. Intra-network relationships (networks within each microhabitat type) were based on network co-occurrence analysis of OTUs with a relative abundance >0.001. Each node represents a bacterial or fungal taxonomic unit, and connections represent statistical significance at *p* < 0.01. The “Hmisc” package in R (Zhong et al., 2022) was used to calculate Spearman correlation coefficients at the genus level. Each node represents an independent sample, and connections represent statistical significance at *p* < 0.01. The Spearman test revealed a correlation coefficient of >0.6. Additionally, the number of nodes and edges, average path length, network diameter, cumulative degree distribution, clustering coefficient, and modularity were calculated based on previous studies using the “network()” function in the R package “ggClusterNet.” The network was visualized using Gephi software version 0.9.2. All statistical analyses were performed using R 4.1.2.

## 3 Results

### 3.1 The impact of elevational changes on soil fungal communities

**Figure 2:**
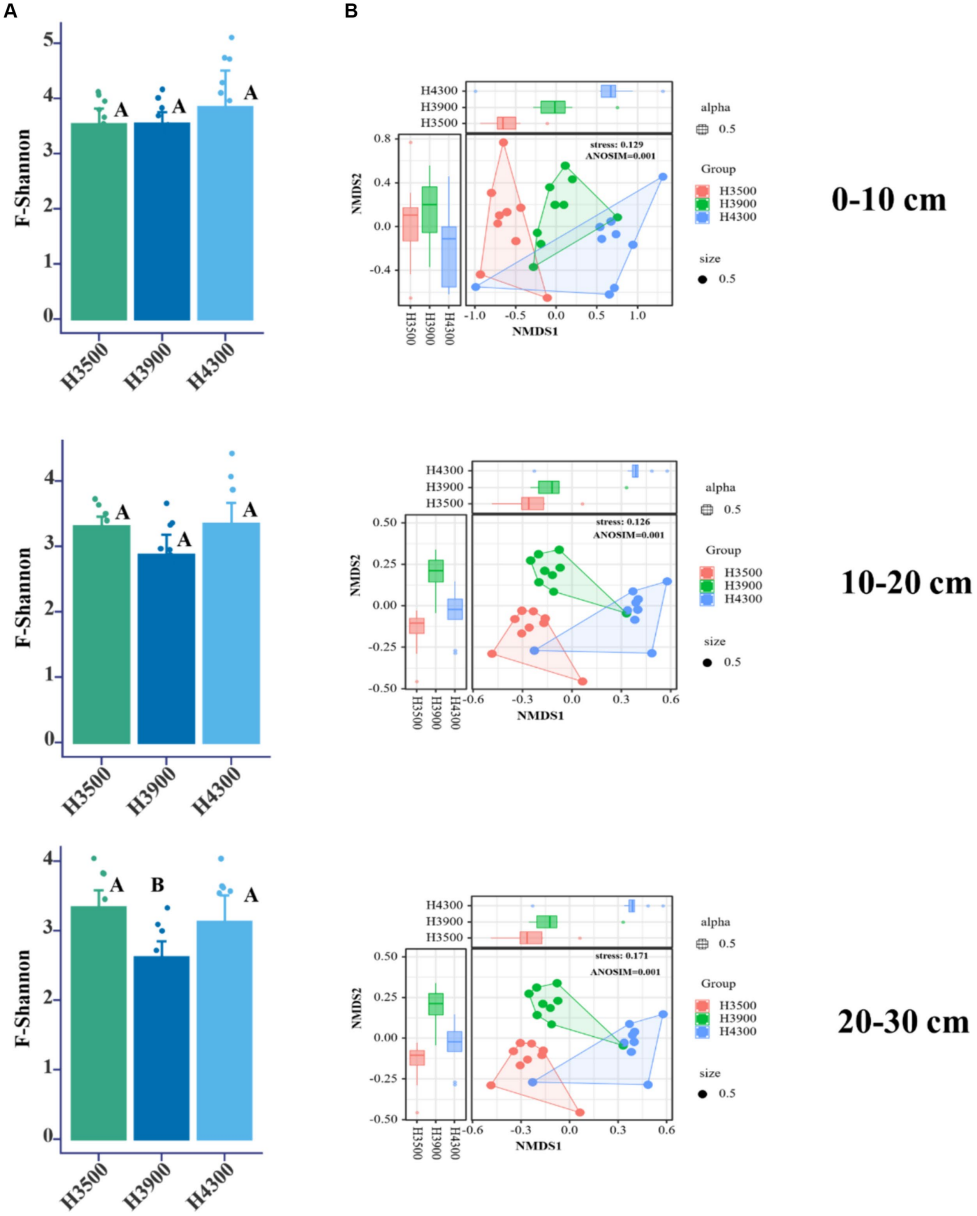
**(A)** Impact of elevational changes on the α-diversity of soil fungal communities in different soil layers. **(B)** Nonmetric multidimensional scaling (NMDS) and analysis of similarity (ANOSIM) based on the Bray–Curtis distance algorithm for soil fungal variability under elevational changes. H3500, H3900, and H4300 represent soils at elevations of 3,500, 3,900, and 4,300 m, respectively; 0–10, 10–20, and 20–30 cm represent soil depths of 0–10, 10–20, and 20–30 cm, respectively.

This study found no significant changes in the α-diversity (Shannon index) of fungal communities in the 0–10 and 10–20 cm soil layers with elevation (Figure 2A, *p* > 0.05). However, in the 20–30 cm soil layer, the α-diversity (Shannon index) of fungi at an elevation of 3,900 m was significantly lower (Figure 2A, *p* < 0.05) than that at elevations of 3,500 and 4,300 m. Based on the Bray–Curtis distance algorithm, NMDS analysis was performed on the OTU abundance table, and ANOSIM was used to further analyze differences in soil microbial communities at different elevations. The results showed that elevation changes significantly affected the fungal community structure in all soil layers (0–10, 10–20, and 20–30 cm) (*p* < 0.05, Stress < 0.2, Figure 2B), and the differentiation of soil fungal community structure became highly significant with increasing soil depth and elevation.

**Figure 3:**
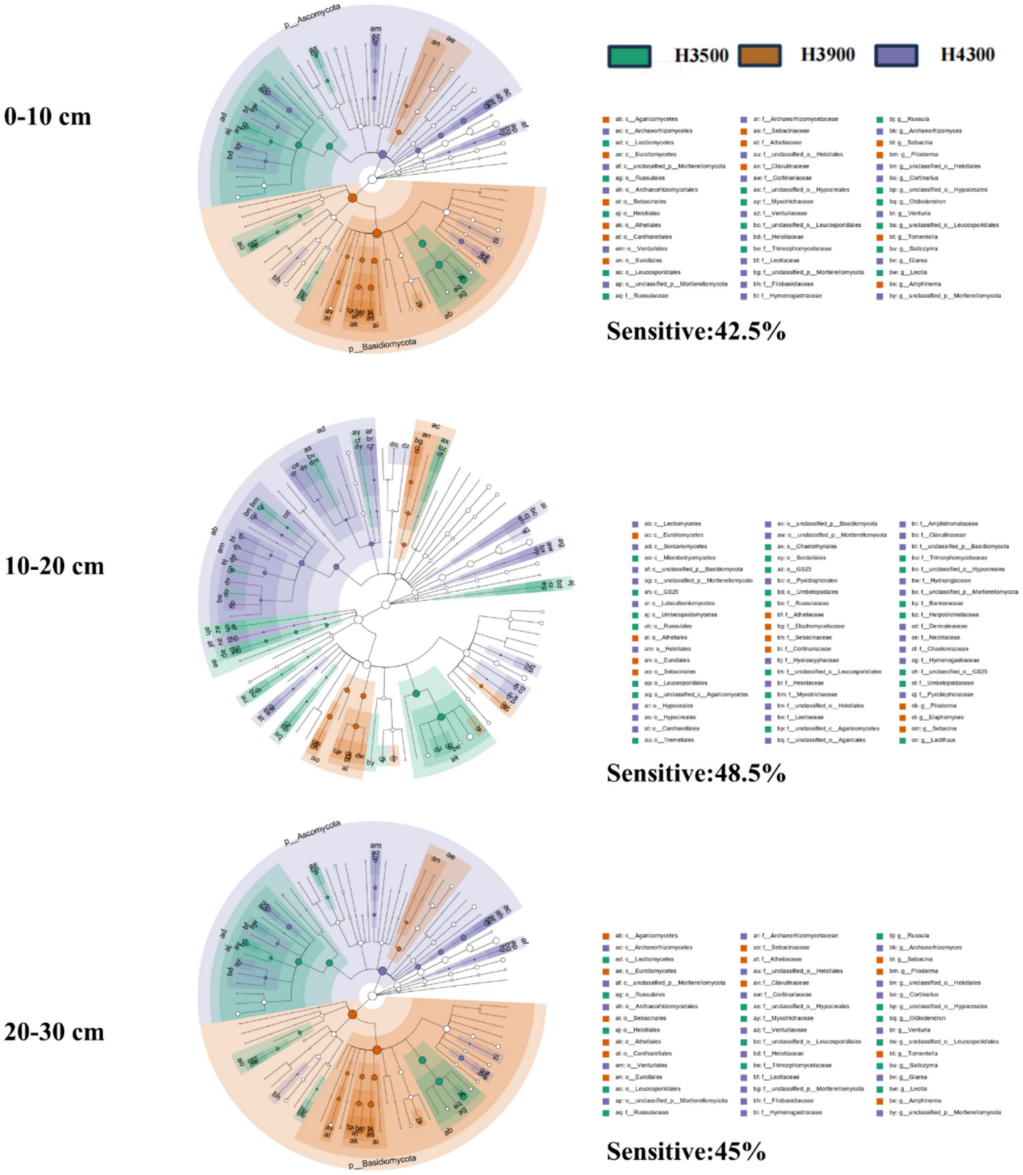
Enriched fungal community biomarkers in various soil layers at different elevations. Taxonomic groups with significant abundance differences among various elevations are represented by colored dots. From the inside to the outside, the six rings of the cladogram denote the kingdom, phylum, class, order, family, and genus. “Sensitive” indicates the proportion of microorganisms with significant differences in relative abundance among different elevations.

Linear discriminant analysis (LDA) effect size (LEfSe) was employed to identify different biomarkers with LDA scores >3, indicating the relative abundance of core microorganisms that exhibit significant changes under environmental disturbances. LEfSe analysis revealed that the relative abundance of fungal communities in all soil layers (0–10, 10–20, and 20–30 cm) changed with elevation. Specifically, the sensitivity of fungal biomarkers in the 10–20 cm (Sensitive: 48.5%) and 20–30 cm (Sensitive: 45%) soil layers to elevation changes was higher than that in the 0–10 cm soil layer (Sensitive: 42.5%) (Figure 3), indicating that 10–20 and 20–30 cm soil layers soil fungal communities are more sensitive to elevation changes. At the phylum level, the relative abundance of *Basidiomycota* and *Ascomycota* exceeds that of other fungi in the 0–10 cm layer. However, as the soil depth increases to 10–20 cm and 20–30 cm, the Basidiomycota predominantly dominates (Supplementary Figure S1).

### 3.2 The impact of environmental variables on soil fungal communities

**Figure 4:**
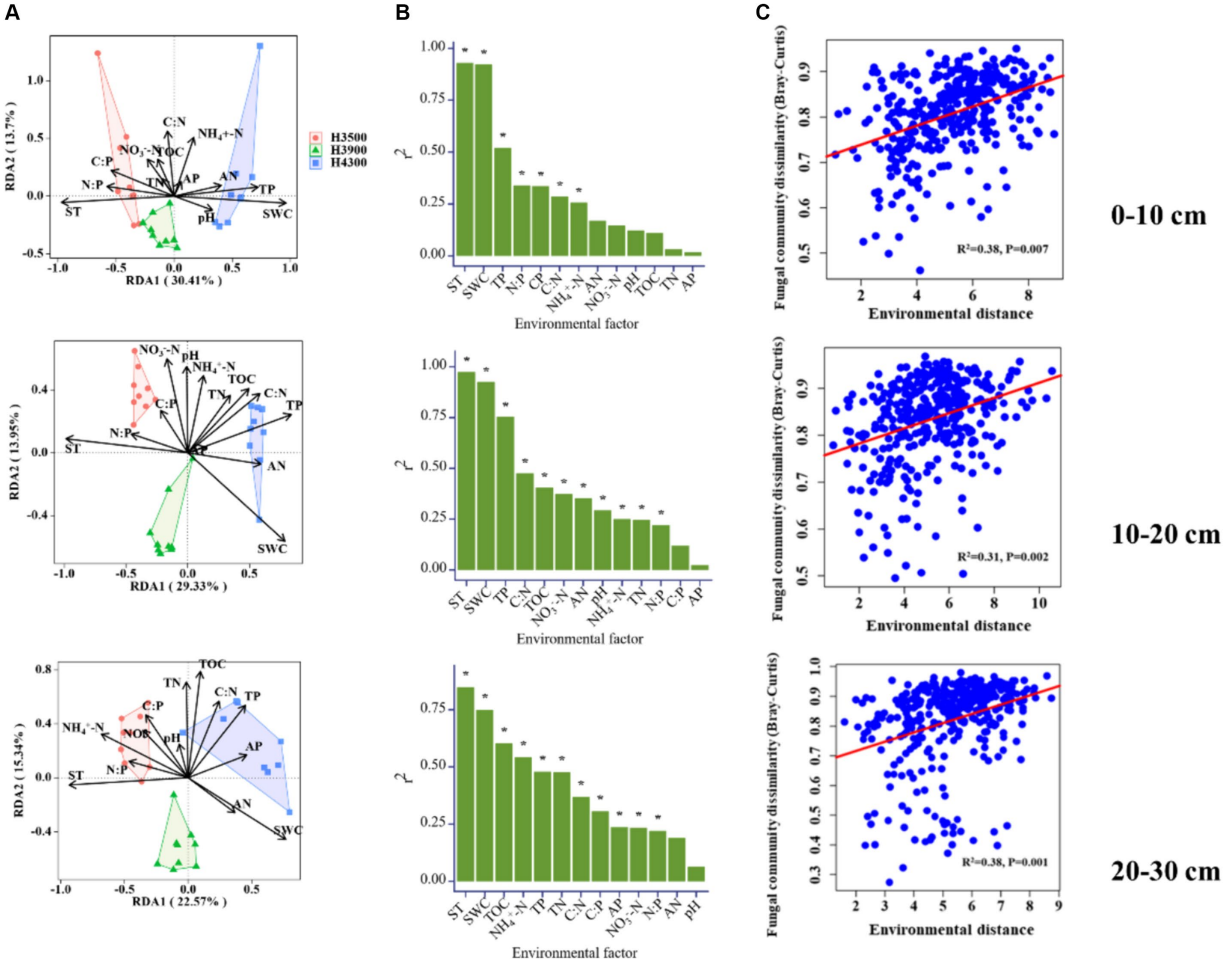
**(A)** Ordination diagram of Redundancy Analysis (RDA) used to determine the relationship between fungi and soil physicochemical properties (indicated by black arrows); **(B)** Diagram showing the amount of variation in community structure due to environmental factors; **(C)** Relationship between environmental distance and fungal community differences (based on Bray–Curtis or Sorensen distances) in soil samples. The solid line represents the fitted linear regression model. TOC, soil total organic carbon; TN, soil total nitrogen; TP, soil total phosphorus; AN, soil available nitrogen; AP, soil available phosphorus; NH_4_^+^ − N, soil ammonium nitrogen; NO_3_^−^–N, soil nitrate nitrogen; SWC, soil water content; ST, soil temperature and pH.

**Figure 5:**
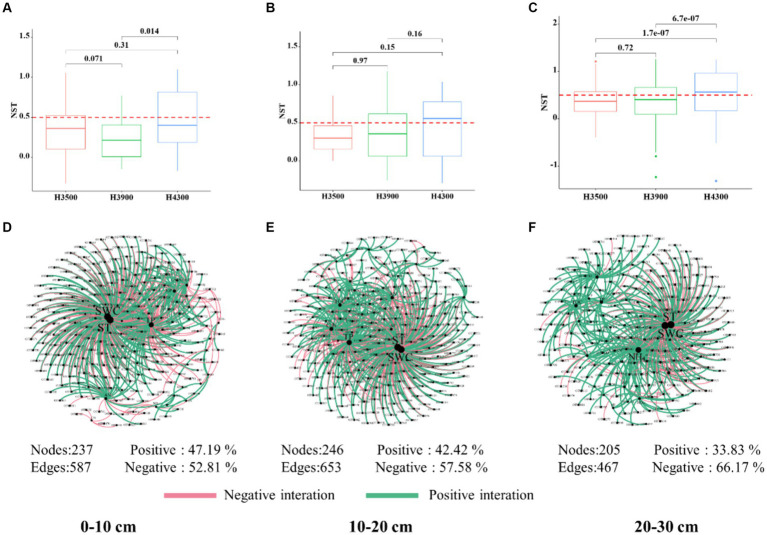
Impact of elevation changes on the assembly process of fungal communities in different soil layers. Normalized stochasticity ratio (NST) at elevations for 0–10 cm **(A)**, 10–20 cm **(B)**, and 20–30 cm **(C)** soil layers. Values above and below the NST threshold of 50% indicate stochastic and deterministic processes, respectively. Taxon–environment factor component networks for microbial communities in 0–10 cm **(D)**, 10–20 cm **(E)**, and 20–30 cm **(F)** soil layers, where node size is proportional to the number of connections. Edges represent strong and significant correlations (*p* < 0.05). The red and green edges indicate positive and negative interactions between two individual nodes, respectively.

The results of the Mantel test showed that ST, SWC, and TP mainly contribute to the soil fungal communities in the 0–10 and 10–20 cm soil layers (Figures 4A,B), while ST, SWC, TOC, and NH_4_^+^ affect soil fungal communities in the 20–30 cm soil layer. In addition, the effect of environmental distance on fungal communities in all soil layers is significant (*p* < 0.05, Figure 4C). The taxon–environment network illustrates the relationship between environmental factors and microbial communities. The results indicated that SWC, ST, and TP were the most closely related factors to microbial communities in the 0–10 and 10–20 cm soil layers (Figures 5E,F), while SWC, ST, and NH_4_^+^ were the most closely related factors to microbial communities in the 20–30 cm soil layer (Figure 5G). Therefore, ST and SWC were identified as the main driving factors for changes in fungal community structure.

### 3.3 The impact of elevation changes on the assembly process of soil microbial communities

In the 0–10 cm soil layer, the NST values of fungi at elevations of 3,500, 3,900, and 4,300 m were < 0.5 (Figures 5A–C), indicating the dominance of deterministic processes. In the 10–20 and 20–30 cm soil layers, the NST values of fungi at elevations of 3,500 and 3,900 m were also <0.5, suggesting the predominance of deterministic processes. However, the NST value of fungi was >0.5 at an elevation of 4,300 m (Figure 5C), indicating that stochastic processes played a leading role in the assembly of fungal communities.

### 3.4 The impact of elevation changes on the co-occurrence network of soil microorganisms

**Figure 6:**
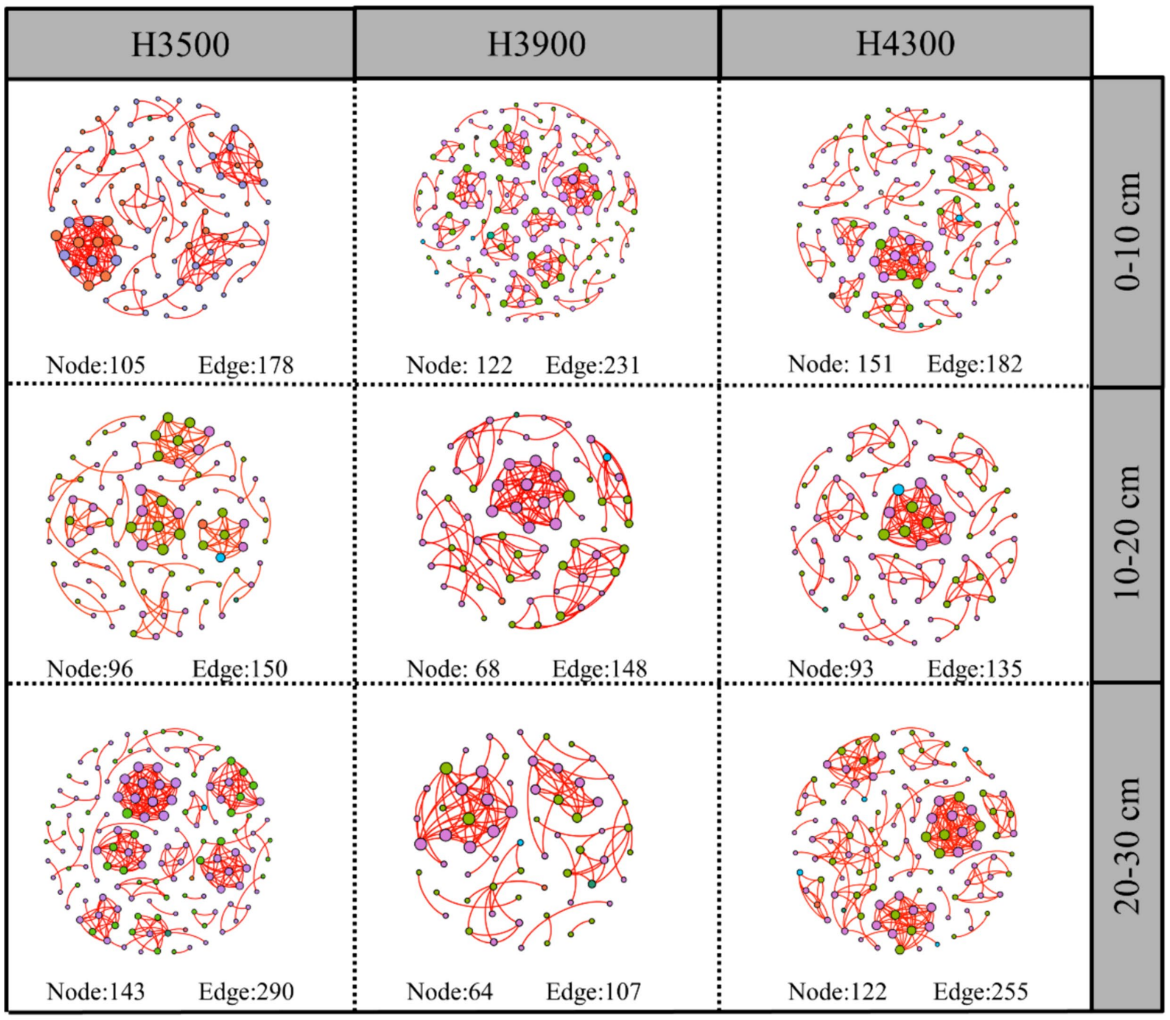
Impact of elevation changes on fungal networks at different soil depths. Each node represents a fungal taxonomic unit at the genus level, and the size of the node is proportional to the relative abundance of all OTUs. The size of each node is proportional to the relative abundance of the corresponding taxonomic unit, and network nodes with the same degree are randomly assigned the same color. Red and blue indicate positive and negative correlations, respectively. Edges represent significant correlations between two corresponding taxa (*r* > 0.6, *p* < 0.01). The thickness of each edge is proportional to the corresponding correlation coefficient. H3500, H3900, and H4300 represent soils at elevations of 3,500, 3,900, and 4,300 m, respectively; 0–10, 10–20, and 20–30 cm represent soils at depths of 0–10, 10–20, and 20–30 cm, respectively.

The complexity of the network was evaluated by assessing its size (number of nodes), number of edges, graph density, network diameter, average clustering coefficient (the degree of node clustering), degree of clustering, and average path length. Network analysis in this study showed that the soil fungal network in the 0–10 cm soil layer at an elevation of 3,500 m had fewer network nodes, graph density, and total network edges compared to those at elevations of 3,900 and 4,300 m (Supplementary Table S1). This finding indicates that the network complexity was lowest at an elevation of 3,500 m, and the complexity of the soil fungal network increased with elevation (Figure 6). In the 10–20 cm soil layer, the soil fungal network at an elevation of 3,900 m had fewer network nodes, graph density, and total network edges compared to those at an elevation of 3,500 m (Supplementary Table S1). This finding indicates that the network complexity at an elevation of 3,500 m was lower than that at 3,900 m. Compared to the fungal network at an elevation of 3,500 m, no significant change was observed in the number of network nodes at 4,300 m, but the total number of network edges was lower (Figure 6). In the 20–30 cm soil layer, the soil fungal network at an elevation of 3,500 m had more network nodes, graph density, and total network edges compared to those at elevations of 3,900 and 4,300 m. Overall, the complexity of the soil fungal network decreased with increasing elevation.

## 4 Discussion

### 4.1 Effects of elevation changes on soil fungal diversity and community structure

We found that in the pure forest of *Abies georgei* var. smithii in the study area, no significant difference was observed in soil fungal α-diversity with elevation changes, which was different from the first hypothesis. This difference may be attributed to the relatively uniform litter types and similar soil nutrient contents at different elevations (Supplementary Figures S2, S3). However, elevation changes still significantly influenced the soil fungal community structure, which was consistent with the studies by Ren et al. (2018) and Siles et al. (2016). High-elevation areas are generally subject to harsher environmental conditions, such as a cold climate, low nutrient availability, and reduced plant biomass, all of which can affect microbial activity (Margesin et al., 2009; D’Alò et al., 2021). In this study area, the *Ascomycota* and *Basidiomycota* phyla emerge as the dominant fungal groups within the microbial community, performing pivotal roles in decomposing organic matter and rhizosphere sediments within the ecosystem (Ma et al., 2022). Notably, in high-elevation environments such as the Sygera Mountains, the significance of fungi in decomposing recalcitrant organic carbon compounds is further underscored by the accumulation of extracellular substances on the 0–10 cm soil layer, which stems from the prevailing low temperatures and sluggish decomposition processes (Treseder et al., 2016). Consequently, both *Ascomycota* and *Basidiomycota* occupy crucial positions in these ecosystems, indispensable for maintaining the cycling of nutrients and the overall health of the soil.

Soil temperature and soil water content were identified in the current study as the main driving factors of soil fungal community structure, and changes in elevation significantly altered the fungal community structures in all soil layers, which aligns with the research findings of Li J. et al. (2023) pertaining to fungal communities within the Qinghai-Tibet Plateau. With increasing elevation, the increase in soil water content may deteriorate the fungal living environment, which, in turn, affects fungal ecological strategies or inhibits oxygen supply to aerobic microorganisms, eventually resulting in differences in fungal communities (Zhao et al., 2022). Meanwhile, the decrease in temperature limits the capability of microorganisms to adapt to extreme environments, leading to changes in the relative abundance of temperature-sensitive fungi and the gradual replacement of dominant fungal groups (Zhao et al., 2024). In addition to temperature and water content, nutrient content is also a crucial factor affecting microbial communities (Xie et al., 2020). This study found that TP and TOC also influence fungal community structure at different elevations. As an energy source and constituent element for fungi, changes in TOC levels affect the elevational distribution of soil fungi by influencing metabolism (Yang et al., 2014). TP, which is involved in fungal metabolic activities and the synthesis of cellular structures, is an essential nutrient for microbial growth, and variations in its content and availability also affect microbial community structure (Pan et al., 2021).

Our study found an interesting phenomenon, that is, the sensitivity of fungi in the 10–20 and 20–30 cm soil layers to elevation changes was significantly higher than that in the 0–10 cm soil layer, which was consistent with the studies by Xu et al. (2023), and aligned with the third hypothesis. This alignment might be due to the typically low nutrient content in 10–20 and 20–30 cm soil layers, which is primarily attributed to the gradual depletion of these resources by plant roots during the process of absorbing nutrients from10–20 and 20–30 cm soil layers and the relatively slow subsequent replenishment of nutrients (Xu et al., 2023). Therefore, fungi living in deep soils may face highly severe resource limitations, increasing their sensitivity to changes in soil environmental conditions. Second, soil water content is one of the key factors affecting fungal characteristics (Liu et al., 2020). Changes in water content in deep soils can significantly impact fungal communities. Specifically, excessively high soil water content can reduce nutrient availability, inhibit the decomposition process of organic matter, and potentially trigger the physiological responses of fungi to environmental stress, thereby affecting their growth and metabolic activities (Wang et al., 2018; Na et al., 2019). These factors work together to increase the sensitivity of 10–20 and 20–30 cm soil layers fungi to environmental changes. Additionally, fungi in 10–20 and 20–30 cm soil layers may largely depend on stable organic matter and root exudates as nutrients. However, these substances may undergo remarkable changes due to climate change (Yu et al., 2024). Therefore, the structure and function of 10–20 and 20–30 cm soil layers fungal communities may be highly vulnerable to impact when external climatic conditions such as temperature and precipitation patterns change (Ma et al., 2022).

### 4.2 The impact of elevation changes on the assembly process of soil fungal communities

The redistribution of resources available to microorganisms can be attributed to differences in the environment and soil characteristics across different elevations. This process may aid microorganisms in adapting to local conditions and altering the assembly patterns of their spatial distribution (Li J. et al., 2023). However, an interesting phenomenon emerged in this study: despite elevation changes, the assembly process of fungal communities in the 0–10 cm soil layer remained relatively stable and was primarily driven by deterministic processes. This finding confirms previous research on subtropical forests (Zhou et al., 2023) and validates the second hypothesis.

Deterministic processes encompass ecological selection pressures exerted by abiotic and biotic factors that influence biological fitness (Vellend et al., 2014). In pristine or relatively undisturbed ecosystems, deterministic processes often predominate (Ning et al., 2020). Interspecies interactions and environmental adaptation are crucial in influencing community structure and maintaining biodiversity. The 0–10 cm soil layer demonstrates an area of extremely high biological activity in the soil. Plants attract diverse microbial communities in the rhizosphere and endorhizosphere environments through root exudates, symbiotic relationships, and close associations with decomposers (Cordovez et al., 2019; Fitzpatrick et al., 2020). These subterranean microorganisms play an indispensable role in assisting plants in coping with various environmental stresses, such as drought and nutrient deficiencies (Lozano et al., 2021; Puy et al., 2022). Therefore, the assembly of fungal communities in this particular soil layer is strongly influenced by deterministic processes, such as interspecies interactions and environmental filtering, resulting in a relatively stable community structure.

We found that the assembly process of fungal communities gradually shifted from deterministic to stochastic processes with increasing elevation. This discovery is consistent with previous research findings from Changbai Mountain (Kang et al., 2023). This transition may stem from the combined effects of various environmental factors and ecological processes. In 0–10 cm soil layer, higher environmental heterogeneity exposes soil fungi to a more diverse array of environmental filters, thereby strengthening the dominant role of deterministic processes in community assembly (Vellend et al., 2014). However, the influence of environmental selection on fungal communities gradually diminishes with increasing soil depth, and other factors begin to dominate the assembly process (Li et al., 2020). Studies have shown that environmental factors and soil nutrients play a jointly determinative role in the assembly of microbial communities (Jiao et al., 2022; Wang et al., 2022). The decomposition of litter is the primary energy source for soil fungi (Crowther et al., 2019). In high-elevation regions, the decomposition rate of litter decreases due to low temperatures, leading to a relatively high accumulation of surface litter. This study found that soil nutrient content at an elevation of 4,300 m was significantly higher in the 10–20 and 20–30 cm soil layers compared to elevations of 3,900 and 3,500 m. This result is consistent with previous research, which indicates that stochastic assembly increases with nutritional conditions, while deterministic processes are more associated with low-nutrient conditions (Zhou et al., 2014; Wan et al., 2021). Therefore, the shift in fungal community assembly patterns in the 10–20 and 20–30 cm soil layers may reflect differences in response and adaptation strategies of deep soil fungi to environmental changes.

### 4.3 Influence of elevation variation on the complexity of soil fungal networks

As a crucial environmental factor, elevation plays a vital role in influencing the distribution and structure of soil fungal communities. With an increase in elevation, the temperature gradually decreases, and soil moisture, nutrient status, and closely related plant community structure also change accordingly (Ma et al., 2022; Fu et al., 2023b). These environmental factors jointly affect the growth, reproduction, and distribution patterns of soil fungi, leading to dynamic adjustments in the complexity of fungal community networks. Fungal communities in different soil layers exhibit opposite responses to environmental changes. As the topsoil that directly interacts with the atmospheric environment, the 0–10 cm soil layer is strongly influenced by climatic factors such as temperature, humidity, and sunlight, as well as plant root activities (Chen et al., 2017; Gao et al., 2021; Yang et al., 2023). Especially at high elevations, the difficulty of decomposing surface litter increases (Pugnaire et al., 2023), providing a unique living space for fungal communities. Therefore, as elevation increases, drastic changes in these environmental factors may lead to an increase in fungal community network complexity to adapt to highly complex and variable environmental conditions.

During the study of soil layers at 10–20 and 20–30 cm depths, a decreasing trend in the complexity of soil fungal community networks was observed with increasing elevation. This finding is consistent with the research conducted by Chen W. et al. (2022) on the Qinghai–Tibet Plateau and confirms the second hypothesis. Climate variables, particularly temperature and precipitation, have considerable impacts on the complexity of microbial networks at the continental scale (Zhang et al., 2018). According to ecological metabolic theory, microbial metabolic activity increases in warm environments (Che et al., 2019). Therefore, the growth rate and metabolic activity of fungi may be suppressed, leading to reduced soil fungal activity and interactions among fungi, eventually resulting in a reduction in network complexity. Furthermore, soil moisture content is another crucial influencing factor. Water can alter soil permeability and the solubility of soil substrates, affecting soil microbial respiration and material diffusion (McCulley et al., 2007). In addition to climatic factors, changes in fungal community network complexity may also be related to species composition and interactions within fungal communities at different elevations (Fu et al., 2023b). With increasing elevation, fungal species adapted to low-elevation environments may gradually decrease or disappear, while those adapted to high-elevation environments may increase. The increase in fungal species could lead to adjustments in competition and symbiotic relationships within the fungal community, thereby influencing the complexity of the fungal community network.

## 5 Conclusion

This study extensively investigated the soil fungal communities of the alpine forest in Sygera Mountain, southeast Tibet, particularly focusing on their responses to elevation changes at different soil depths. The results indicate that the relative abundance of fungal communities in 10–20 and 20–30 cm soil layers are significantly more sensitive to elevation changes than those in 0–10 cm soil layer. As elevation increases, the diversity and structure of fungal communities in deep soil underwent notable changes. Specifically, the 0–10 and 10–30 cm soil layers exhibited opposing trends in the complexity of fungal networks in response to elevation changes, further emphasizing the pivotal role of soil depth in fungal communities’ response to elevation variations. Additionally, this study found that with the increase in elevation, the assembly mechanism of fungal communities in 10–20 and 20–30 cm soil layers gradually shifted from deterministic to stochastic, while the assembly mechanism in 0–10 cm soil layer remained relatively stable. This study enhances our understanding of how soil fungi at varying depths respond to elevational changes in alpine forests, offering invaluable scientific evidence for assessing and predicting the dynamic changes of soil microorganisms in the carbon cycle under future climate change scenarios. Given soil microorganisms’ active involvement in organic matter decomposition and transformation, along with their pivotal role in stabilizing organic carbon and regulating soil carbon storage and turnover, this research significantly aids in grasping the functionality of terrestrial carbon sinks and developing effective strategies to address climate change.

## Data availability statement

The raw sequencing data can be found in the NCBI Sequence Read Archive under the accession number PRJNA1140373.

## Author contributions

BZ: Data curation, Formal analysis, Investigation, Methodology, Software, Validation, Visualization, Writing – original draft. SZ: Data curation, Formal analysis, Investigation, Methodology, Software, Validation, Visualization, Writing – original draft. JiaL: Conceptualization, Funding acquisition, Project administration, Resources, Supervision, Writing – review & editing. FF: Investigation, Software, Visualization, Writing – original draft. LG: Investigation, Visualization, Writing – original draft. JieL: Investigation, Visualization, Writing – original draft. YZ: Investigation, Visualization, Writing – original draft. YL: Investigation, Visualization, Writing – original draft. GC: Investigation, Visualization, Writing – original draft. GZ: Software, Visualization, Writing – original draft.

## Footnotes

^1^http://huttenhower.sph.harvard.edu/lefse/
