# Supplementary material for: Elevational distribution patterns and drivers factors of fungal community diversity at different soil depths in the Abies georgei var. smithii forests on Sygera Mountains, southeastern Tibet, China
Source: Front Microbiol. 2024 Aug 9;15:1444260. doi: 10.3389/fmicb.2024.1444260 (PMC11342059; doi:10.3389/fmicb.2024.1444260)
Supplement: Supplementary file 1 [file Data_Sheet_1.pdf]

Supplementary materials

Table S1 Fungal network topology in soil layers along different elevational gradients.

Network Topology	soil depth								
	0-10 cm			10-20 cm			20-30 cm		
elevation	3500 m	3900 m	4300 m	3500 m	3900 m	4300 m	3500 m	3900 m	4300 m
node	105	151	122	96	68	93	143	64	122
edge	178	231	182	150	148	135	290	107	255
average degree	3.39	3.06	2.984	3.125	4.53	2.903	4.056	3.344	4.18
network diameter	2	2	3	2	2	1	2	1	4
graph density	0.033	0.02	0.025	0.033	0.065	0.032	0.029	0.053	0.035
modularization	0.778	0.931	0.875	0.872	0.809	0.812	0.87	0.786	0.874
Average clustering coefficient	0.943	0.963	0.889	0.972	0.981	1	0.963	1	0.968
Average path length	1.073	1.025	1.052	1.02	1.007	1	1.01	1	1.102

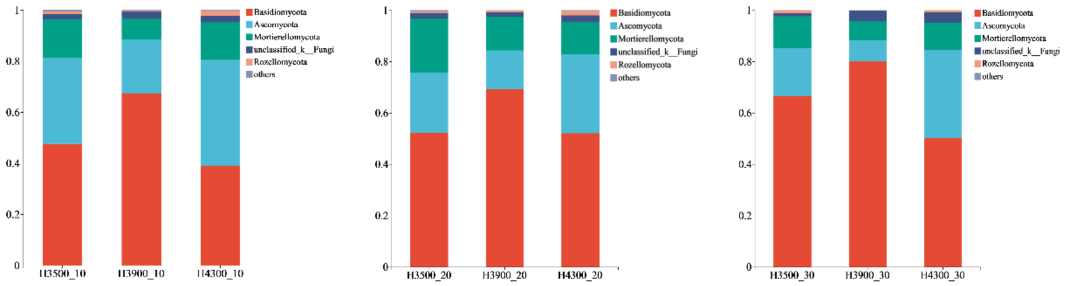

Figure S1 Impact of elevation changes on the composition of fungal communities at different soil depths.

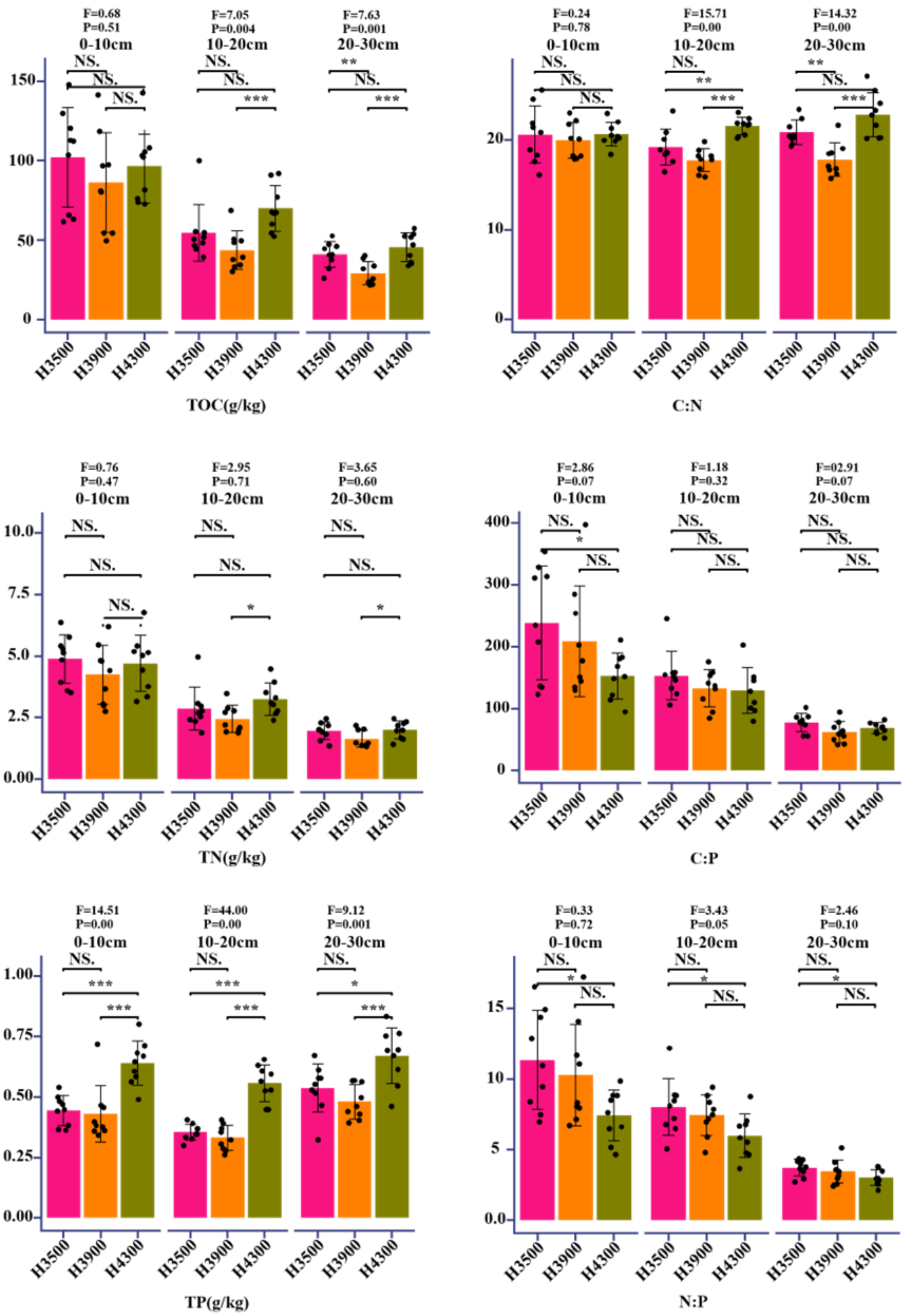

Figure S2 Contents of total organic carbon (TOC), total nitrogen (TN), total phosphorus (TP), and their stoichiometric ratios at different elevations and soil depths. *P<0.05; **P<0.01.

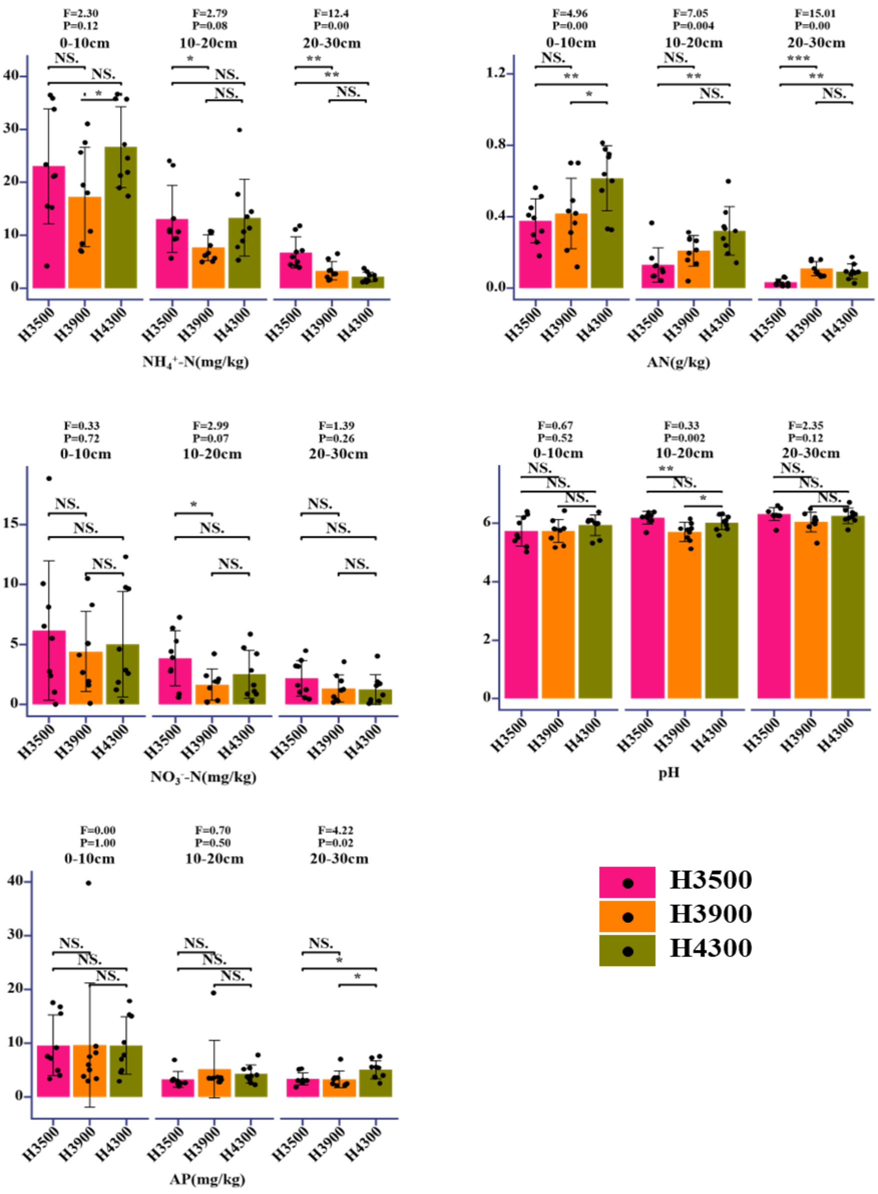

Figure S3 Ammonium nitrogen (NH_4^+-N), nitrate nitrogen (NO_3^--N), available phosphorus (AP), available nitrogen (AN), and soil pH at different elevations and soil depths. **P*<0.05; ***P*<0.01.
